# Functional and effective whole brain connectivity using magnetoencephalography to identify monozygotic twin pairs

**DOI:** 10.1038/s41598-017-10235-y

**Published:** 2017-08-29

**Authors:** M. Demuru, A. A. Gouw, A. Hillebrand, C. J. Stam, B. W. van Dijk, P. Scheltens, B. M. Tijms, E. Konijnenberg, M. ten Kate, A. den Braber, D. J. A. Smit, D. I. Boomsma, P. J. Visser

**Affiliations:** 1 Alzheimer Center and Department of Neurology, Neuroscience Campus Amsterdam, VU University Medical Center, Amsterdam, The Netherlands; 2 Department of Clinical Neurophysiology and Magnetoencephalography Center, VU University Medical Center, Amsterdam, The Netherlands; 3 Department of Biological Psychology, VU University Amsterdam, Amsterdam, The Netherlands; 4 Department of Psychiatry, Academic Medical Center, Amsterdam, The Netherlands

## Abstract

Resting-state functional connectivity patterns are highly stable over time within subjects. This suggests that such ‘functional fingerprints’ may have strong genetic component. We investigated whether the functional (FC) or effective (EC) connectivity patterns of one monozygotic twin could be used to identify the co-twin among a larger sample and determined the overlap in functional fingerprints within monozygotic (MZ) twin pairs using resting state magnetoencephalography (MEG). We included 32 cognitively normal MZ twin pairs from the Netherlands Twin Register who participate in the EMIF-AD preclinAD study (average age 68 years). Combining EC information across multiple frequency bands we obtained an identification rate over 75%. Since MZ twin pairs are genetically identical these results suggest a high genetic contribution to MEG-based EC patterns, leading to large similarities in brain connectivity patterns between two individuals even after 60 years of life or more.

## Introduction

Inter-individual variability in functional brain connectivity has been associated with inter-individual differences in measures of cognitive functioning^1^, gender^2^, ageing^3, 4^ and the presence of brain pathology^5^. Despite the observation that resting-state networks (RSNs) have a topographic core that is homogeneous between individuals^6–9^, recent papers have shown that it is possible to reliably identify single-subjects based on their functional connectivity patterns as measured with functional magnetic resonance imaging (fMRI)^10, 11^. Therefore, these patterns can be regarded as ‘functional connectivity fingerprints’ (FCFs) or connectivity profiles. In this study, we considered four different ways of defining connectivity: three undirected (functional) and one directed (effective). Functional and effective connectivity capture two different aspects of interaction between time-series. The former evaluates the statistical interdependency between two time-series without giving any information about the influence of one time-series on the other, whereas effective connectivity captures the influence of one signal on the other^12^.

We investigated whether the functional (FC) or effective (EC) connectivity patterns of one monozygotic (MZ) twin could be used to identify the co-twin among a sample MZ twin pairs. MZ twins arise from a single fertilized egg and share all their genetic material, i.e. have the same genetic background^13^. We therefore aimed to test the similarity on the FCFs and effective connectivity fingerprints (ECFs) using magnetoencephalography (MEG).

Previous studies in twins support the genetic influence on whole-brain summary statistics such as the average functional connectivity across all brain regions or measures of functional brain network topology as assessed by electroencephalography (EEG), MEG and fMRI^14–21^ with estimates of heritability varying between 42 and 72%. Importantly, twin studies analyzing the contribution of genetics, shared environment, and unique environment to functional connectivity by comparing overlap in connectivity within MZ and dizygotic (DZ) twin pairs concluded that the concordance between twins was mainly due to genetic factors and not the shared environment^14–16^. This indicates that resemblance in brain connectivity within MZ twins can be attributed to genetics, rather than a shared environment.

Here, we investigated the resemblance between MZ twin pairs in functional and effective connectivity, i.e. going beyond whole-brain summary statistics^14–22^. If genes indeed have a major effect on connectivity profiles, then MZ twin pairs should be identifiable from these profiles. Furthermore, to better understand the genetic effect on the subject-specific connectivity profiles, we extended our analysis by removing the connectivity patterns that are shared between individuals (‘functional or effective connectivity backbone’), and attempted to identify MZ twin pairs on the basis of only their unique functional or effective connectivity patterns. The functional or effective connectivity backbone most likely underlies highly conserved functions^23^, whereas the residual functional or effective connectivity patterns express the inter-individual variability and the influence of genetic and shared environmental factors on this latter is still unknown.

We hypothesized that source-level MEG FCFs and ECFs, computed from resting-state data, enable reliable identification of MZ twin pairs and can therefore be regarded as fingerprints. We assessed the discriminative effectiveness of MEG fingerprints computed with three functional connectivity measures (phase lag index^24^, amplitude envelope correlation^25–27^ with and without signal-leakage correction) and one effective connectivity estimate (directed phase transfer entropy^28, 29^) in order to capture different coupling modes^30^: phase relations, amplitude envelope relations, and directed interactions, respectively.

## Results

**Figure 1:**
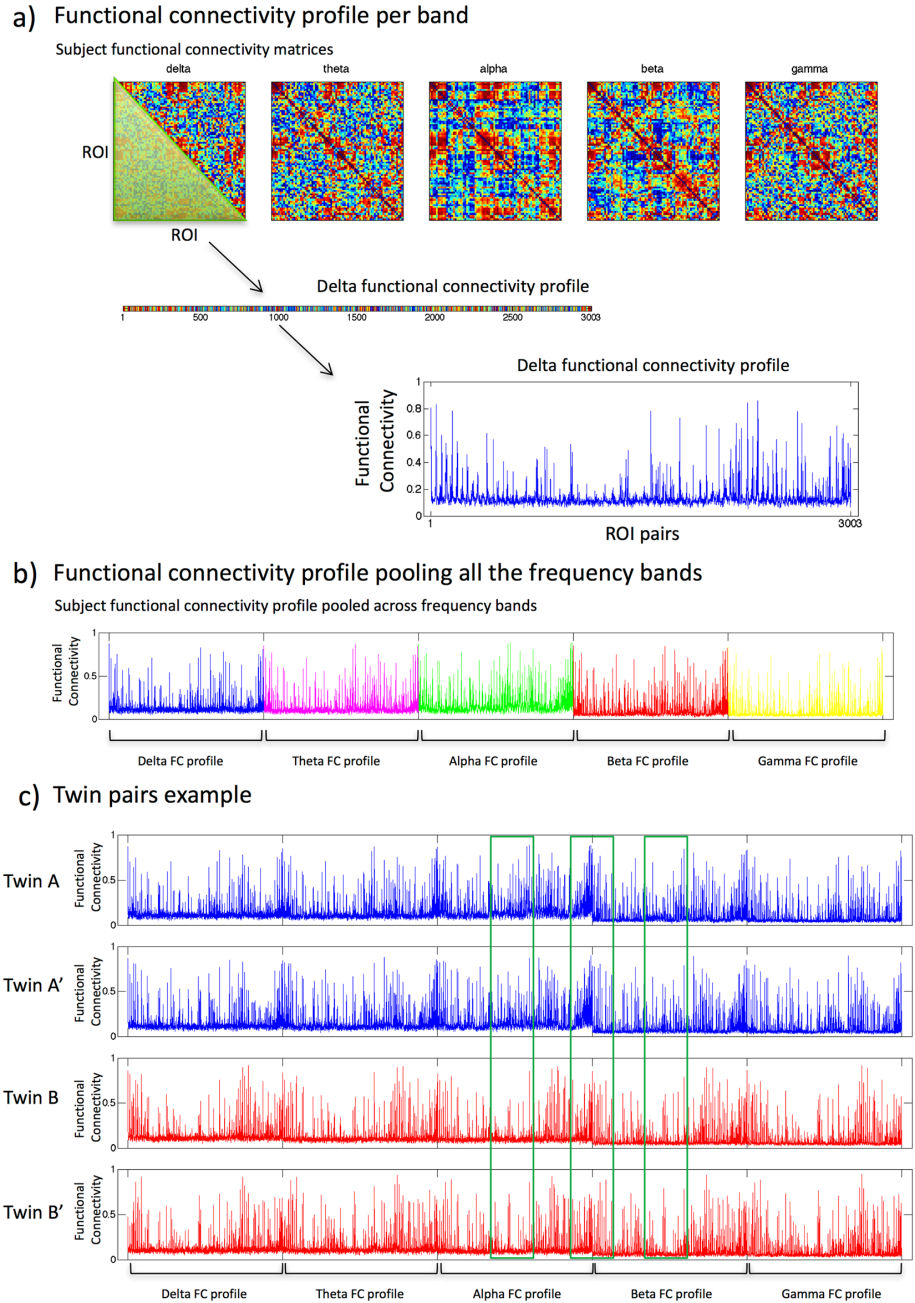
Example of functional connectivity fingerprints. For every subject, and for every frequency band, a FC matrix was computed using a FC measure (**a**). Every matrix contains ranked values for visualization purposes. From every FC matrix the lower triangular entries, consisting of the pair-wise connectivity between all possible combinations of ROIs (i.e. $(\frac{78\ast 77}{2})=3003$), were extracted. These entries correspond to the FCF for a frequency band. An example of a FCF in the delta band is shown in the bottom plot in (**a**) where the y-axis shows FC values and the x-axis are the ROI pairs. A FCF was computed for every frequency band and these were pooled together (delta FCF in blue, theta FCF in magenta, alpha FCF in green, beta FCF in red and gamma FCF profile in yellow) to obtain a single global FCF (**b**), which was used for the identification analysis. An example of global FCFs for two different twin pairs ((A-A′) and (B-B′)) is shown in (**c**). Visual comparison of the FCFs suggest that within a twin pair FCFs are more similar than between unrelated subjects (see green ellipsoids in (**c**)). The same strategy was used to obtain ECFs.

We analyzed data from 32 monozygotic twin pairs (64 subjects in total) from the Netherlands Twin Register (NTR^31^) who take part in the EMIF-AD preclinAD study (see methods section) on predictors for Alzheimer’s disease biomarkers in cognitively healthy elderly subjects. MEG data consisting of 5 minutes no-task eyes-closed resting-state recordings were source-reconstructed to 78 cortical regions (regions of interest, ROIs) of the automated anatomical labeling (AAL) atlas^32^. Functional (FC) and effective (EC) connectivity was assessed between all pairs of regions with Amplitude Envelope Correlation (AEC), Amplitude Envelope Correlation with leakage correction (AEC-c), Phase Lag Index (PLI) and directed Phase Transfer Entropy (dPTE) in 5 frequency bands (delta (0.5–4 Hz), theta (4–8 Hz), alpha (8–13 Hz), beta (13–30 Hz), and lower gamma (30–48 Hz)). For every subject we obtained an average FC or EC matrix for every FC or EC measure in every frequency band, resulting in FCFs and a ECF for every frequency band (see Fig. 1a).

In order to combine the information from different bands, for every FC and EC measure we pooled the FCF or ECF of the different frequency bands in one global FCF or ECF (see Fig. 1b), which was used for the identification analysis. The similarities between FCFs or ECFs were quantified with Spearman’s correlation coefficients that were subsequently converted into distance scores. The identification analysis consisted of an iterative process, in which at every step a subject was selected and his or her FCF or ECF was compared with the FCFs or ECFs of all other subjects. If the lowest distance score was obtained for the subject’s co-twin we considered it a successful outcome (a match), otherwise a miss. The rate of successful identification across all individuals was calculated as the ratio between the number of successful outcomes and the total number of subjects. Statistical significance of the observed success rate was assessed by means of permutation testing^33^.

Conventionally, statistical inference of functional connectivity patterns are performed at group level, which has shown reliable connectivity patterns^6, 9, 25, 26, 34, 35^. However, within these patterns it is possible to identify at least two components: a common pattern that is shared among subjects (low variance across subjects) and unique patterns (high variance across subjects) representing the individual connectivity patterns that contribute to the inter-subject variability^36, 37^. We applied singular value decomposition (SVD) to the individual FCFs and ECFs (independently for every FC or EC measure) in order to disentangle these two components and repeated the identification analysis using the pooled FCFs and ECFs from which the shared pattern was removed.

In order to understand the relative contribution of each frequency band to the identification performances we repeated the identification analyses using the FCFs or ECFs of each band separately, again with and without the shared pattern.

### Subject characteristics

Thirty-two monozygotic twin pairs (21 female pairs) with a mean age of 68.13 (±7.93 standard deviation) years participated in the study. All participants were cognitively normal and scored 25 or higher on the Mini Mental State Examination (mean and standard deviation 28.84 ± 1.16). The mean duration of education was 15.08 years (±4.43 standard deviation). Mean education score was 5.16 (±0.95 standard deviation) based on the Dutch Classification System by Verhage (1964) consisting of a 7-point scale, ranging from primary education not finished (1) to master degree (7).

### Identification analyses using FCFs or ECFs

**Figure 2:**
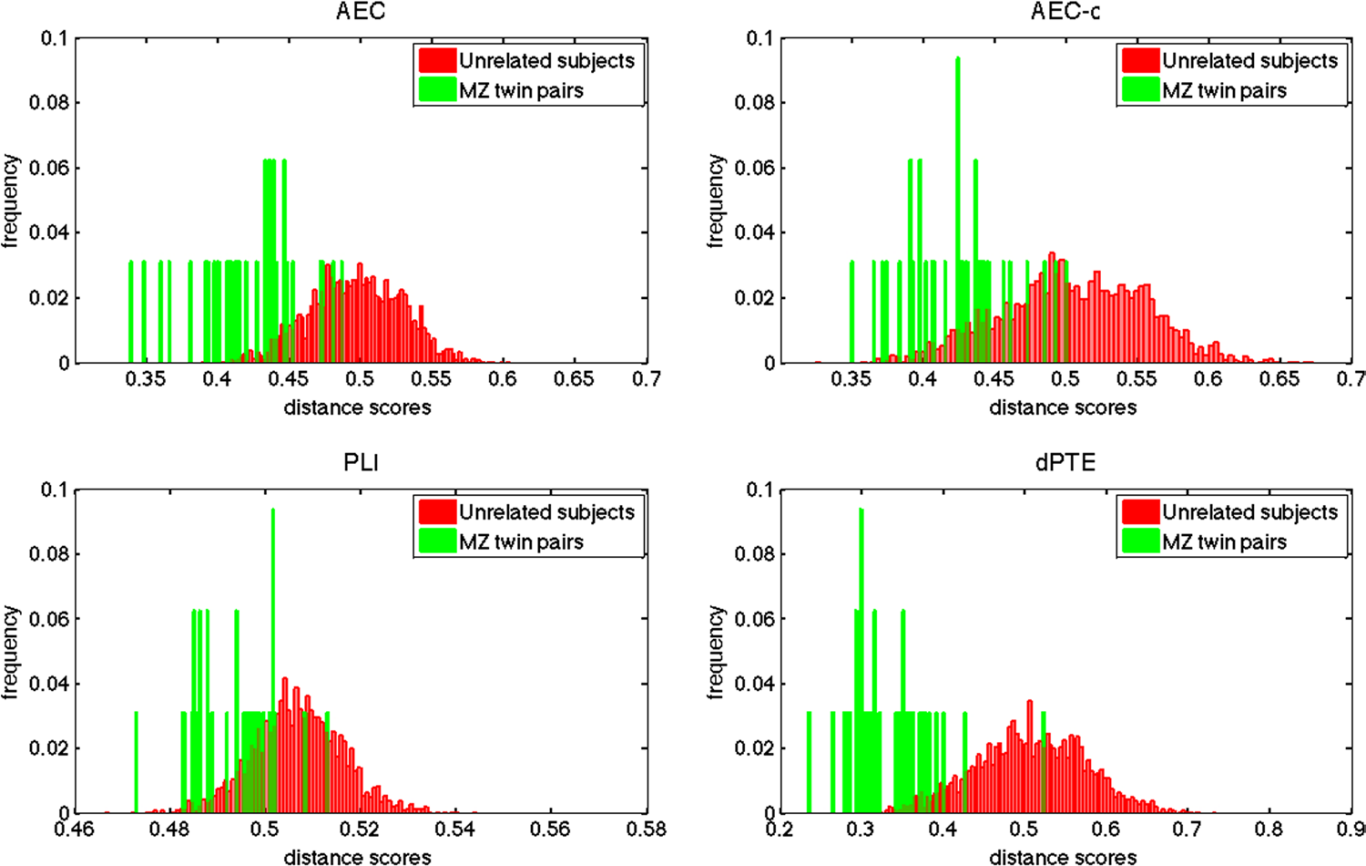
Distance score histograms for MZ twin pairs and for genetically unrelated subjects. For every connectivity measure (AEC, AEC-c, PLI and dPTE) the distance score distributions are displayed. These distance scores were computed using the global FCFs or ECFs after the removal of the shared pattern. Note the differences in scales for the x-axes. Also note that score distributions obtained with dPTE were further apart compared to the distributions obtained when using other connectivity metrics.

Identification success rates obtained combining the information from all frequency bands are shown in Table 1 for both the original data and after removal of the common pattern. For every measure, the highest significant success rate was obtained after removal of the common pattern: dPTE success rate was over 75% ($49/64=76.6 \% ,\,p=0.001)$, AEC was higher than 50% ($34/64=53.1 \% ,\,p=0.001)$, AEC-c was close to 40% ($24/64=37.5 \% ,\,p=0.001)$ and PLI was around 9% ($6/64=9.4 \% ,\,p=0.002)$. Success rates obtained with the common pattern included were lower, namely: dPTE ($37/64=57.8 \% ,\,p=0.001)$, AEC ($23/64=35.9 \% ,\,p=0.001)$ and AEC-c ($15/64=23.4 \% ,\,p=0.001)$, with the PLI result not being significant. Figure 2 shows distance score histograms for MZ twin pair and genetically unrelated subjects for the case where the common pattern was removed. Note that for the dPTE the score distributions for twin pairs and unrelated pairs (identification rate 76.6%,) were further apart compared to the distributions obtained when using other FC measure metrics (see Figure S1 in the supplementary information file for all the fingerprint comparisons).

**Table 1:** Twin identification success rate using the global FCF or ECF based on different measures.

	*FC profile pooled across bands*	
FC	*original*	*SVD*
AEC	35.9%***	53.1%***
AEC-c	23.4%***	37.5%***
PLI	3.1%	9.4%**
dPTE	57.8%***	76.6%***

For every subject the global FCF or ECF was obtained combining the subject’s FCF or ECF computed for the individual frequency band. The success rate for every FC and EC connectivity measure is reported: Amplitude Envelope Correlation without (AEC) and with correction (AEC-c), directed Phase Transfer Entropy (dPTE) and Phase Lag Index (PLI). Success rate based on the original data, as well as after removal of the common pattern across subjects (using SVD), are given. The asterisks represent the significant values after permutation testing: p-value ≤ 0.05(*), p-value ≤ 0.01(**) and p-value ≤ 0.001(***).

For every FC and EC measure, twin pair identification was also performed using the FCF or ECF for each individual frequency band in order to assess the discriminative power of every frequency band alone. Results are shown in Table 2 both for the original data and after the removal of the common pattern. Identification success rates varied across FC and EC measures and frequency bands: with the original data the highest significant success rate, although moderate, was obtained for dPTE in the alpha band ($23/64=35.9 \% ,\,p=0.001)$. Success rates higher than 25% were observed for dPTE in the beta ($17/64=26.6 \% ,\,p=0.001)$ and theta ($16/64=25.0 \% ,\,p=0.001)$ bands, and for AEC without correction in the beta band ($17/64=26.6 \% ,\,p=0.001)$.

**Table 2:** Twin identification success rates for different FC or EC measures in individual frequency bands: Amplitude Envelope Correlation without (AEC) and with correction (AEC-c), directed Phase Transfer Entropy (dPTE) and Phase Lag Index (PLI).

FC	Delta		Theta		Alpha		Beta		Gamma	
	*original*	*SVD*	*original*	*SVD*	*original*	*SVD*	*original*	*SVD*	*original*	*SVD*
AEC	6.2%*	23.4%***	12.5%***	29.7%***	15.6%***	40.6%***	26.6%***	48.4%***	14.1%***	29.7%***
AEC-c	4.7%	9.4%**	4.7%	10.9%***	14.1%***	31.2%***	18.8%***	26.6%***	4.7%	6.2%*
PLI	1.6%	3.1%	4.7%	1.6%	7.8%**	4.7%	4.7%	6.2%*	4.7%	9.4%**
dPTE	3.1%	1.6%	25.0%***	21.9%***	35.9%***	40.6%***	26.6%***	37.5%***	12.5%***	15.6%***

The success rate based on the original data, as well as after removal of the common pattern across subjects (using SVD), are given. The asterisks represent the significant values after permutation testing: p-value ≤ 0.05(*), p-value ≤ 0.01(**) and p-value ≤ 0.001(***).

Although we also observed significant identification rates for the corrected AEC (alpha and beta bands), these rates were lower compared to when using AEC without leakage correction. The only significant success rate for PLI was in the alpha band ($5/64=7.8 \% ,\,p=0.004)$.

Across measures and frequency bands the identification performances generally increased after removal of the common pattern. The best results for most measures were observed in theta, alpha and beta bands. For AEC without correction the success rate improved for all the frequency bands, with the highest value ($31/64=48.4 \% ,\,p=0.001)$ in the beta band and the lowest value ($15/64=23.4 \% ,\,p=0.001)$ in the delta band. Likewise, the success rates for AEC with leakage correction, with highest and lowest value in the alpha ($20/64=31.2 \% ,\,p=0.001)$ and gamma band (4/64 = 6.2%, *p* = 0.018), respectively. Again, PLI success rates were generally low compared to the other FC and EC measures, with significant, yet low, success rates only in the beta ($4/64=6.2 \% ,\,p=0.014)$ and gamma ($6/64=9.4 \% ,\,p=0.002)$ band. The significant success rates for dPTE increased especially for the alpha ($26/64=40.6 \% ,\,p=0.001)$ and beta ($24/64=37.5 \% ,\,p=0.001)$ bands.

In summary, the best identification was obtained for the alpha and beta band after removal of the common pattern, especially for the dPTE and uncorrected AEC.

## Discussion

In this study, we investigated the resemblance between twins from 32 monozygotic pairs using MEG FC and EC patterns. We showed that it is possible to identify which MZ twins belong to the same pair from a pool of subjects exploiting the EC patterns. The high success rate obtained (over 75%) indicated that MEG EC patterns act as a functional fingerprint. Despite the observation that resting-state FC patterns are shared between subjects^6, 9, 25, 26, 34, 35^, we observed, on top of this common pattern, that they also provided reliable information to identify MZ twin pairs from unrelated pairs. These observations indicated that MEG EC patterns are genetic traits.

Although previous studies (see refs 38 and 39 for reviews) have demonstrated high heritability of FC, such analyses were performed on summary whole-brain statistics and not on the full FC or EC patterns and so it was unclear whether this would allow for MZ twin pair identification.

Heritability of FC during a resting-state paradigm has been previously assessed with fMRI in specific sub-networks such as default-mode network^19^ (h^2^~40%) and extended to other resting-state networks^20^. Moreover network topology has also been shown to be heritable^18, 21, 22^ (h^2^ 42%-60%). Heritability of FC has been observed in EEG studies^14–17^, however these studies adopted either a whole-brain statistic (i.e. averaging across all pair-wise connectivity values or an overall network topological measure) or per-electrode statistic (i.e. averaging connectivity per recording site). Here we showed that EC patterns carried sufficient heritable information to allow for an ~75% correct identification.

FC and EC can be measured in different ways, which is likely to influence results. Therefore, we investigated the influence of different FC and EC measures on twin identification. Envelope correlation measures (like AEC and AEC-c) and phase-based measures (like PLI) are related to distinct coupling mechanisms^30^, characterized by different dynamics and possibly involved in different cognitive processes^30^. We further extended the study of neuronal synchronization considering causal relationships (effective connectivity) between neuronal populations. Understanding what is the influence that a neuronal population exerts on another one is crucial to decipher cognitive processes^12^. The use of a directed measure such as dPTE^28, 29^ allowed the estimation of such causal relationships (in Granger and Wiener terms).

The best identification rates using the pooled FCFs or ECFs (both with the original data and after the removal of the shared pattern, identification rates 57.8% and 76.6% respectively) were obtained with dPTE suggesting that the directionality of interactions provided reliable information for the identification. We speculate that both structural and functional aspects play a role in these results. Recently, the mesoscale structural connectome of the mouse was disclosed^40^ and a striking finding was the asymmetry in the connectivity profile (i.e. difference in in- and out-going connections). This asymmetry was a key concept exploited by Henriksen colleagues^41^ to build a growing model that successfully reproduces the mouse connectome with its directed connectivity. We argue that asymmetry of connections may be a fundamental property of mammalian connectome^42, 43^ even though a comprehensive blue-print about afferent and efferent connections is lacking in humans^44^. Measures such as dPTE may be more prone to detect the influence of such anatomical asymmetries than undirected measures and this extra information is beneficial for identification. Indeed, structural connections influence FC and EC patterns however they do not coincide. Different complex dynamical phenomena can arise on top of a fixed structural network and there is theoretical evidence^45^ that effective connectivity^12^ may result from self-organization of brain rhythm activity. In addition, the advantages of transfer entropy (TE) measures in detecting the complex dynamics has recently been reported^46^. Estimates of connectivity patterns through the dPTE probably have richer information content that allow outperforming undirected FC measures (i.e. AEC, AEC-c and PLI) for identification.

Each measure has different strengths and weaknesses. Specifically, the activity coming from a neuronal source can be detected by different sensors (field spread), resulting in spurious estimates of FC between sensors. This problem is mitigated, yet not completely solved^47^, in source-space, where it is commonly referred as signal-leakage^48, 49^. Interpretation of FC estimates is therefore problematic if metrics are used that do not address this problem. AEC does not correct for such spurious estimates of functional connectivity, while AEC-c, PLI and dPTE do address this problem directly^24, 25, 28, 50^. Although handling this problem aids interpretation, our results showed that identification rate is generally lower when correcting for field spread. For example, we observed a decrease from an identification rate of 53.1% using AEC without correction to 37.5% after the correction (AEC-c) using the FC profiles without the shared patterns. The same it is true for the original data: identification rate decreased from 35.9% with AEC to 23.4% with AEC-c. Moreover, PLI showed only one significant result with a success rate of 9.4%. Together, these findings for AEC, AEC-c and PLI suggested that zero-lag interactions provide valuable information for identification. The drop in performance for AEC after leakage correction and the poor results for PLI could be caused by ignoring the zero-lag interactions, which might have included true interactions. Conversely, as reported by Colclough and colleagues^51^, the higher performance for uncorrected connectivity metrics could be related to the fact that these measures may reflect trivial properties arising from the spatial configuration of sources, which hinder interpretability.

Moreover, the decrease in performance for AEC after correction could be related to the signal to noise level: power in orthogonalized time-series is an order of magnitude lower than before orthogonalization^50, 51^. Furthermore, part of the success of uncorrected AEC could be due to the fact that band power itself is highly heritable^52^.

In Table 2, it can be observed that uncorrected AEC (after removal of the shared pattern) shows higher (or equal) identification rate compared to dPTE for all frequency bands, however when all bands are combined dPTE outperforms AEC (76,6% vs 53,1%). An explanation for this result may be found in the observation that dPTE contains potentially more independent information in different frequency bands than uncorrected AEC. Figure S2 shows that the connectivity matrices for uncorrected AEC are fairly similar across frequency bands, whereas for the dPTE matrices differ more across the frequency bands. This may influence the overall identification rate when pooling the bands together: in the case of AEC redundant information is pooled together, whereas potentially independent information is pooled when using dPTE, leading to a better identification rate.

The removal of the shared pattern across subjects from the individual FCFs or ECFs improved the identification rates for each connectivity measures. Recently, Hawrylycz^23^ and colleagues demonstrated that a shared FC pattern across individuals relate to consistent gene expression signatures. Likely, this FC pattern underlies highly conserved functions. Since we aimed to analyze at individual level data, we decided to discard this shared FC or EC pattern across individuals in order to assess the influence of familial factors on the residual FC or EC patterns. It might be argued that the removal of the shared pattern across subjects from individual functional connectivity profiles reduces the generalizability of the results because this transformation is group-dependent. However, the recent literature on the consistency and repeatability of MEG functional connectivity patterns at group-level^9, 37, 51^ supports the use of this approach in our study. We showed that also the FC and EC patterns that are specific to the individual are strongly influenced by familial factors.

Recently, Finn and colleagues^10^ reported high identification rates with fMRI fingerprints (~95% and ~99% using whole brain and sub-network fingerprints respectively in resting-state condition) without performing any removal of a shared FC pattern. However, our results support that the removal of this common pattern enhances identification rate. The high identification rate without removal of a shared pattern in Finn’s study may be related to two main differences with the present study: first, they matched the same subject while we aimed to identify one subject using the fingerprint of its twin; second, the higher dimension of their fingerprint (35778 entries in the feature vector obtained from 268 ROIs) compared to our fingerprint (15015 entries in the feature vector obtained from 78 ROIs times 5 frequency bands). The effect of the fingerprint size is confirmed by a result reported as well in Finn’s study. They recomputed the identification rates using a different parcellation scheme with fewer ROIs (68 ROIs, 2278 entries in the feature vector) and the identification rates dropped (~89% and ~75% using whole brain and sub-network fingerprints respectively in resting-state condition). We recomputed as well the identification rates using a parcellation scheme with a higher resolution (see Tables S3 and S4). By using a higher resolution atlas, the identification rates for both the original data and after the removal of a shared pattern improved or remained stable for every connectivity measure (compared to using the AAL atlas).

Although the identification rate generally improved with the high resolution parcellation scheme, the best identification rate (dPTE after removal of the shared pattern, pooled across frequency bands) did not improve (76.6%). This observation can probably be ascribed to the resolution of the beamformer approach in combination with resting-state MEG data, which represents an upper bound on the independent information that can be reconstructed.

Other than for PLI, using the frequency-specific FCFs independently for the identification analysis gave the best results for the alpha and beta band (both with and without the shared patterns). These results are in accordance with previous EEG studies on heritability^15–17, 52^ and a recent study in which the high reliability of FC in these bands was reported^37^. In addition, although the whole power spectrum was reported to be heritable^52, 53^, power in alpha and beta bands exhibited higher values of heritability. We speculate that this could be related to a distinct genetic origin of different brain rhythms^54^. A recent work by Richiardi *et al*.^55^ emphasizes the relationship between FC patterns and genes, showing how genes associated to ion channels and synaptic function control the spatial organization of functional resting-state networks in fMRI.

### Limitations

In our study we could not disentangle genetic from shared environment sources of variance^56^ because the study included MZ twins only. However, previous research^14–16^ has shown that shared environmental factors are negligible in FC estimates.

Our results are related to an elderly population and so future studies should investigate how these results would generalize to younger individuals.

Another limitation of this study is the small number of subjects, which could have inflated identification rates. Even though the best identification rate (~75%) may seem low compared to other studies^10^ we would like to emphasize that in our study we matched different subjects, which is a harder problem than identifying the same subject between different observations (i.e. recordings), as was the case in these previous reports. We did not consider different source reconstruction strategies for the solution of the inverse problem. Recently, it has been shown^57^ that results in source space are affected by different choices during the analysis pipeline (i.e. inverse model, type of connectivity measure and different software implementations). Hence, although our results were obtained using a tried-and-tested analysis pipeline^29, 47, 58–61^ that has also been implemented by other groups^62, 63^ future studies should reproduce our findings using alternative approaches.

Finally, it would be interesting to further investigate to what extent different resting-state networks^6–8^ are related to either the shared pattern across individuals or the residual connectivity patterns. This would help to understand which connectivity patterns are related to conserved functions^23^ or inter-individual variability. Recently, it was shown that fronto-parietal networks are mostly related to inter-individual variability, and this improved the identification rate compared to a whole brain approach^10^. However, our main goal was to show, at a global level, if MZ twin identification was feasible using MEG effective and functional connectivity fingerprints. Further studies focusing on the relative influence of different sub-network on the identification rates are desirable.

## Conclusion

We conclude that MEG-based effective connectivity patterns can be considered as fingerprints that are highly specific to individuals, under strong genetic influence, and might be good candidates to study the influence of genetic variation on brain functioning and ultimately inter individual differences in behavior and/or psychopathology.

## Material and Methods

### Subject Information

#### No table of contents entries found

The sample for this study comes from the Netherlands Twin Register and takes part in an ongoing study on heritability of Alzheimer’s disease risk factors in cognitively healthy elderly subjects, which is part of the Innovative Medicine Initiative (IMI) European Information Framework for Alzheimer Disease project (EMIF-AD). The aim of the PreclinAD study is to collect 300 cognitively normal elderly participants with ages ranging from 60 to 100 years. Subjects are recruited from the Manchester and Newcastle Aging Study (MNAS) (n = 100) and, as a twin sub-study, from the Netherlands Twin Register (NTR) (n = 200; 100 monozygotic twin pairs).

### Inclusion criteria

In this study data from the first 32 NTR monozygotic twin pairs enrolled in the PreclinAD study were analyzed. This subset was used because these subjects had an MEG recording available at the time when analysis was performed.

Inclusion criteria were age 60 years and older, Telephone Interview for Cognitive Status modified (TICS-m) > 22^64^; Geriatric Depression Scale (GDS) (15 item) < 11^65^; Consortium to Establish a Registry for Alzheimer’s Disease (CERAD) 10 word list immediate and delayed recall (>−1.5 SD of age adjusted normative data)^66^; Clinical Dementia Rating (CDR) scale of 0 with a score on the memory sub-domain of 0^67^.

Exclusion criteria included neurological and psychiatric diseases such as mild cognitive impairment, brain tumor, brain infection, schizophrenia, bipolar disorder, Parkinson’s disease, epilepsy; other systemic illness/co-morbidity (e.g. thyroid disease, uncontrolled diabetes mellitus, cancer), recreational drug use, alcohol consumption (>35 units per week), and use of medication that may influence cognition (e.g. benzodiazepine, lithium carbonate, antipsychotics including atypical agents, antidepressants, or Parkinson’s disease medicines).

This study and all procedures were carried out in accordance with a protocol approved by the ethical board of the VUmc (Medische Ethische Toetsingscommissie VUmc, project number 2014.210, approval date 2014-11-27). All subjects provided written informed consent.

### Data acquisition

#### Neuropsychological assessment

Subjects underwent extensive neuropsychological testing and questionnaires. The Mini-Mental State Examination was administered to assess cognitive status^68^.

#### MRI acquisition

Anatomical whole brain scans were obtained using a 3.0T MR scanner (Philips Achieva). Isotropic structural 3D T1-weighted images were acquired using a sagittal fast field echo sequence (repetition time = 7.9 ms, echo time = 4.5 ms, flip angle = 8°, 1 mm × 1 mm × 1 mm voxels).

#### MEG acquisition

MEG data were recorded using a 306-channel whole-head MEG system (Elekta Neuromag Oy, Helsinki, Finland) while participants were in supine position inside a magnetically shielded room (Vacuumschmelze, Hanau, Germany). MEG recordings were performed before the MRI scan. Magnetic fields were recorded at a sample frequency of 1250 Hz, with an anti-aliasing filter of 410 Hz and a high-pass filter of 0.1 Hz. The protocol consisted of 5 minutes in a eyes-closed resting-state condition (i.e. not performing any task), followed by 2 minutes in an eyes-open condition, and then again 5 minutes in an eyes-closed condition. Only the first 5 minutes of eyes-closed data were used for the analysis.

The head position relative to the MEG sensors was recorded continuously using the signals from five head-localization coils. The head-localization coil positions were digitized, as well as the outline of the participants scalp (~500 points), using a 3D digitizer (Fastrak, Polhemus, Colchester, VT, USA).

Channels that were malfunctioning during the recording, for example due to excessive noise, were identified by visual inspection of the data, and removed (median = 9, range 2–13) before applying the temporal extension of Signal Space Separation (tSSS) in MaxFilter software (Elekta Neuromag Oy, version 2.2.15)^69–71^. The tSSS filter was used to remove artifacts that SSS without temporal extension would fail to discard, typically from noise sources near the head, using a subspace correlation limit of 0.9 and a sliding window of 10 seconds.

The digitized scalp surfaces of all subjects were co-registered to their structural MRIs using a surface-matching procedure, with an estimated resulting accuracy of 4 mm^72^. A single sphere was fitted to the outline of the scalp as obtained from the co-registered MRI, which was used as a volume conductor model for the beamformer approach described below.

#### Beamforming

An atlas-based beamforming approach^47^ was used to project the MEG data to 78 regions of interest (ROIs) in source-space, using the AAL atlas^32^. For a detailed description we refer the reader to (Hillebrand *et al*. 2016). The broadband (0.5–48 Hz) time-series at sensor level were projected through the normalized broadband beamformer weights for each ROI’s centroid in order to obtain a time-series for each ROI. From these time-series, the first 18 epochs each containing 16384 samples (13.10s), were selected^73, 74^.

These time-series were then downsampled to a sample frequency of 312 Hz (yielding epochs of 4096 samples each) and filtered in classical EEG/MEG frequency bands (delta (0.5–4 Hz), theta (4–8 Hz), alpha (8–13 Hz), beta (13–30 Hz), and lower gamma (30–48 Hz)), using an offline discrete FFT filter that does not distort the phases^75^.

#### Functional and effective connectivity

Pairwise FC and EC was estimated between each of the 78 ROIs for each frequency band using three FC and one EC measure listed below. All four measures are based on the computation on the Hilbert transform to obtain the analytic signal which was used estimate the envelope or the instantaneous phase. The measures used to estimate FC and EC are (see supplementary information file for details):

- Amplitude Envelope Correlation (AEC), which detects amplitude-based coupling among brain signals. AEC captures interactions between two time-series computing the correlation between their envelopes^27^.
- The leakage-corrected Amplitude Envelope Correlation (AEC-c), where time-series were orthogonalized by means of linear regression analysis before estimating functional connectivity with AEC^50^. This correction was performed on the band-filtered time series in time domain with the aim to reduce trivial spurious correlations induced by signal leakage. Orthogonalization of two time series can be done in two directions (i.e. given X and Y as time series, X can be regressed out from Y, and Y can be regressed out from X). For every pair of time series we computed the orthogonalization in both directions, and then we averaged the AEC values computed on the orthogonalized time series for the two directions.
- Phase Lag Index (PLI)^24^ is a measure of the asymmetry of the distribution of phase differences between two time series. It reflects the consistency of phase relations between two time series, avoiding zero-lag (mod π) phase coupling and thereby minimizing the influence of spurious correlation induced by leakage.
- Phase Transfer Entropy (PTE) is a directional phase-based measure that estimates information flow on the basis of the transfer entropy between the time series of the instantaneous phases^28, 76^. We used the implementation of dPTE as described in^29^, which is bounded in the range 0.5 < dPTE_xy_ ≤ 1 when information flows preferentially from a time series X to time series Y. However when information flows preferentially toward X from Y, 0 ≤ dPTE_xy_ < 0.5. In the case of no preferential direction of information flow, dPTE_xy_ = 0.5.

Subsequent analysis steps were performed independently for every FC and EC measure and for all frequency bands.

The calculation of FC and EC measures resulted in 18 (one for each epoch) FC or EC matrices for each subject, which were then averaged per subject. No threshold was applied to the connectivity matrices.

For each subject, a FCF and ECF was extracted from the average FC or EC matrix by vectorizing its lower triangular part not including the diagonal. This vector therefore has 3003 entries $(\frac{78\ast 77}{2})$, which represent the FC or EC values between every pair of ROIs. Usually a directed connectivity matrix will not be symmetrical; however, due to the way dPTE is defined the two triangular parts are trivially related: the dPTE for a pair of regions is computed by normalizing the PTE values by the sum of the PTE values in both directions ($dPTE=\frac{PT{E}_{xy}}{{{\rm{PTE}}}_{{\rm{xy}}}+{{\rm{PTE}}}_{{\rm{yx}}}}$, see supplementary information for details), which forces the upper and lower triangular part of the dPTE matrix to add up to one $(dPT{E}_{xy}=1-dPT{E}_{yx})$. Hence even for the dPTE matrix we considered only the lower triangular part like for the other FC matrices.

We combined for every subject the FCFs or ECFs for the individual frequency bands (independently for every functional connectivity measure). This resulted in a global FCFs or ECFs across bands (15015 entries = 3003 × 5 frequency bands). The global FCF or ECF was then used for the identification analysis using either the FCFs (or ECFs) with or without the shared pattern, and statistical significance was assessed using permutation testing.

#### Twin pair identification using functional or effective connectivity

Spearman’s rank correlation was used as a similarity measure to compare two FCFs or ECFs. In order to deal with negative correlations, similarity scores were transformed into distance scores using the following formula (eq. 1):

1 $$distance\,score=1-\frac{\begin{array}{c}(correlation\,coefficient+1)\end{array}}{2}$$

The identification analysis consisted of an iterative process, in which at every step a subject was selected and its FCF or ECF was compared with the other subjects’ FCFs or ECFs, yielding 63 distance scores. If the lowest distance score was obtained for the subject’s co-twin we considered it a successful outcome (a match), otherwise a miss. We repeated this process for all 64 subjects and we calculated the success rate as the ratio between the number matches and total number of subjects.

In order to assess statistical significance of the observed success rate we used permutation testing. At each iteration we first randomly redefined the relatedness between subjects (i.e. subjects were randomly assigned to be twin pairs), next the identification analysis was performed and the success rate computed. This procedure was repeated 1000 times to build a permuted success rate distribution, and the observed success rate was compared against this distribution to determine the p-value.

#### Singular Value Decomposition

Twin pair identification performances might improve by removing the contribution of the connectivity pattern shared among FC or EC profiles. To this end, all connectivity profiles were pooled in a matrix (**M**_15015×64_) and the singular value decomposition (SVD) of this matrix was computed (eq. 2):

2 $${{\bf{M}}}_{15015\times 64}={{\bf{U}}}_{15015\times 64}\,{{\bf{S}}}_{64\times 64}\,{{{\bf{V}}}^{{\rm{T}}}}_{64\times 64}$$

where **U** is the matrix containing the left singular vectors, **S** contains the singular values, **V** is the matrix containing the right singular vectors, and T is the matrix transpose. The left singular vector is associated with the largest singular value and represents the dominant common pattern shared among the connectivity profiles. Projecting back the matrix of the connectivity profiles without the contribution of the largest singular value and its corresponding left and right singular vectors yielded the connectivity profiles without the shared pattern.

The computation of the dPTE was performed using Brainwave software (BrainWave, version 0.9.150.6; home.kpn.nl/stam7883/brainwave.html), all the other analyses were performed using in-house MATLAB scripts (MATLAB Release 2012a, The MathWorks, Inc., Natick, Massachusetts, United States) and an additional MATLAB plotting script from http://www.mathworks.com/matlabcentral/fileexchange/ (tight_subplot.m).

### Data availability

The data that support the findings of this study are available from the corresponding author upon reasonable request.

## Electronic supplementary material

Supplementary Information
