# Supplementary material for: Functional and effective whole brain connectivity using magnetoencephalography to identify monozygotic twin pairs
Source: Sci Rep. 2017 Aug 29;7:9685. doi: 10.1038/s41598-017-10235-y (PMC5575140; doi:10.1038/s41598-017-10235-y)
Supplement: Supplementary file 1 — Supplementary Information [file 41598_2017_10235_MOESM1_ESM.doc]

# Supplementary Information

**Functional and effective whole brain connectivity using magnetoencephalography to identify monozygotic twin pairs**

M. Demuru^1*^, A.A. Gouw^1,2^, A. Hillebrand^2^, C.J. Stam^2^, B.W. van Dijk^2^, P. Scheltens^1^, B.M. Tijms^1^, E. Konijnenberg^1^, M. ten Kate^1^, A. den Braber^1,3^, D.J.A. Smit^3,4^, D. I. Boomsma^3^, P.J. Visser^1^

^1^Alzheimer Center and Department of Neurology, Neuroscience Campus Amsterdam, VU University Medical Center, Amsterdam, The Netherlands,

^2^Department of Clinical Neurophysiology and Magnetoencephalography Center, VU University Medical Center, Amsterdam, The Netherlands,

^3^Department of Biological Psychology, VU University Amsterdam, Amsterdam, the Netherlands,

^4^Department of Psychiatry, Academic Medical Center, Amsterdam, the Netherlands,

# Functional and effective connectivity formulas and implementation details

## Phase Lag Index (PLI)

The PLI for two time series can be computed using the following formula^1^:


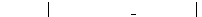
 (1)

where


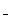
 is the phase difference between the instantaneous phases of the two time series computed using the Hilbert transform;
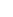
 are the discrete time step where the instantaneous phases are computed;
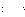
 denotes the average across the time-samples t;
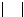
 denotes the absolute value.

## Amplitude Envelope Correlation (AEC)


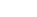
 and
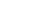
 are two time series after applying the Hilbert transform to two time series *X* and *Y*. The AEC can then be computed following the formula^2^:


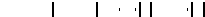
 (2)

and *corr* the Pearson’s correlation coefficient.

## Directed Phase Transfer Entropy

Given a source time series
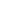
 and a target time series
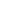
 the transfer entropy
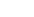
 is given by the following formula^3^:


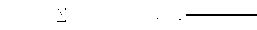
 (3)

where
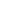
 represent a discrete time point and
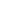
 denotes a delay. The some runs over all time discrete time step
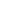
.

If the instantaneous phases are considered as the arguments of the
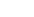
 then the phase transfer entropy (PTE) is obtained^4,5^.

We assume independent probability distributions (for the joint probability term only), so that
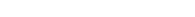
Note that this assumption has no influence on the information flow patterns yet speeds up the computation time^6^.The PTE then becomes:


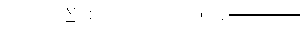
 (4)

where the probabilities are obtained by building the histograms of occurrences of the single, pairs or triplets of the phase estimates. The number of bins in the histograms was set by the following formula^7^ :


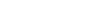
 (5)

the delay
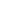
 was set as
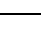
 where
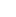
 and
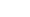
 are the number of samples and the number of regions of interest (ROIs), i.e. number of time series, considered, respectively; and
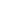
 is the number of times the phases changes sign across time and ROIs.

Finally, the directed phase transfer entropy (dPTE) was computed as


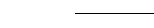
 (6)

which is bounded in the range 0.5 < dPTE_XY_ ≤ 1 when information flows preferentially from a time series X to time series Y. However, when information flows preferentially toward X from Y, 0 ≤ dPTE_XY_ < 0.5. In the case of no preferential direction of information flow, dPTE_XY_ = 0.5.

The dPTE was computes using BrainWave, version  0.9.150.6, which can be downloaded from [home.kpn.nl/stam7883/brainwave.html](http://home.kpn.nl/stam7883/brainwave.html).

# Fingerprint correlations

Figure S1 shows the correlations between the fingerprints for every pair of subjects for different connectivity measures. The common pattern was removed from the global functional connectivity fingerprint (or effective connectivity fingerprint) using SVD. Monozygotic twin pairs are placed in consecutive positions (i.e. (1;2) first pair; (3;4) second pair etc.). The correlations between a subject i and all other subjects can be observed along a row. Hence, the best identification rate is obtained when the highest values (dark red in Figure S1) are obtained next to the diagonal (in 2x2 blocks). In Figure S1 it can clearly be seen that dPTE has the best identification performance.


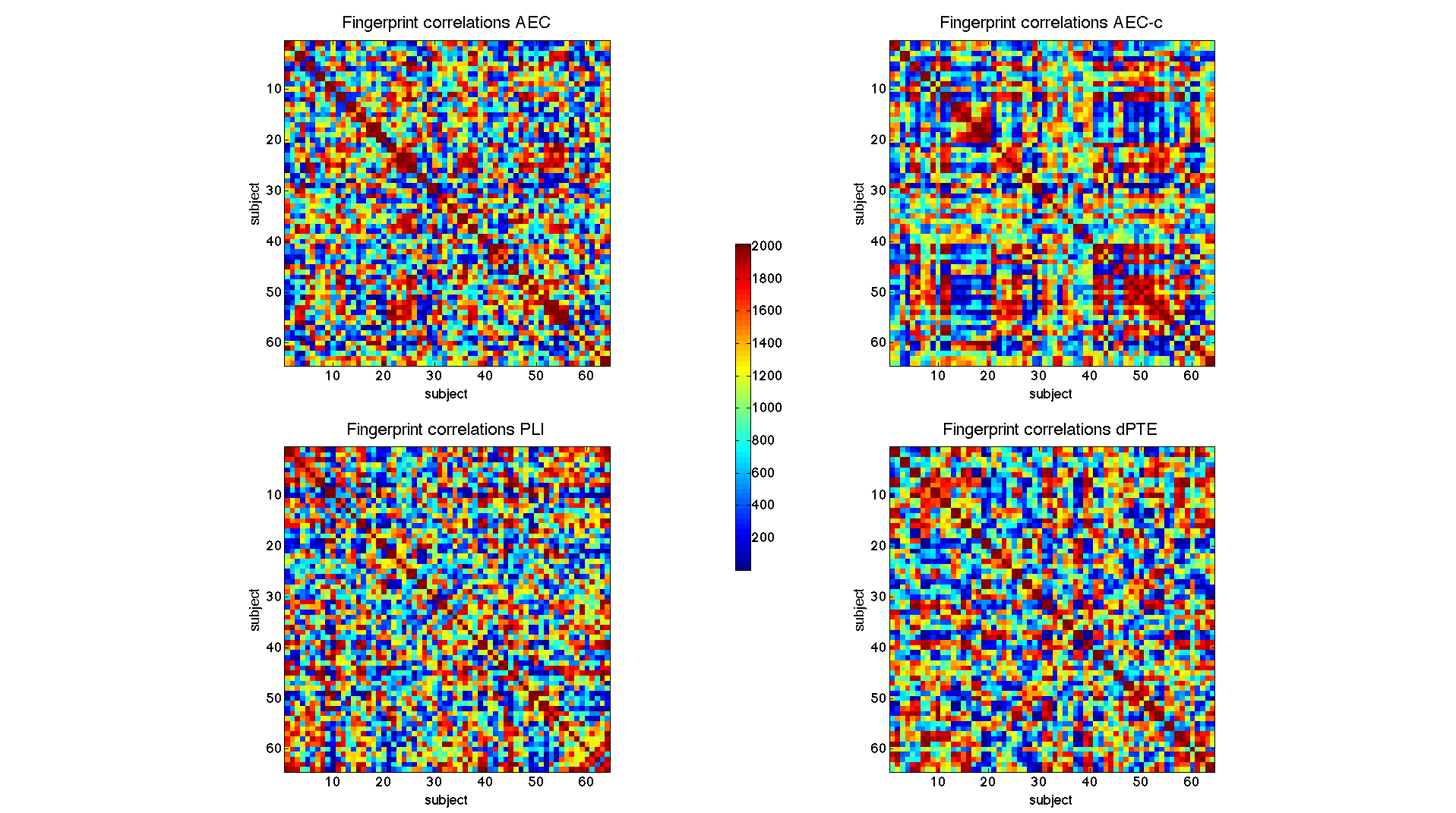


Figure S1 Correlations between the fingerprints for every pair of subjects for different connectivity measures. The fingerprints are relative to the case where the common pattern was removed from the global functional connectivity fingerprint (or effective connectivity fingerprint) using SVD. The values in each cell are ranked. A twin pair is placed in consecutive positions (i.e. (1;2) first pair; (3;4) second pair etc.). Hence, the best identification rate is obtained when the highest values (dark red) are obtained next to the diagonal (in 2x2 blocks).

# MEG Functional connectivity and Effective connectivity patterns

Figure S2 shows the grand average FC matrices for every frequency band. ROIs in the matrices are ordered from left to right hemisphere in the following way: 1-15 indices represent left frontal regions, 16-21 left parietal regions, 22-27 left occipital regions, 28-39 left temporal regions, 40-54 right frontal regions, 55-60 right parietal regions, 61-66 right occipital regions and 67-78 right temporal regions (see Table S2). Different patterns of connectivity can be recognized in the matrices obtained with different connectivity measures.

On visual inspection, the matrices computed with AEC without leakage correction show, for all frequency bands, a pattern where neighboring regions (entries around the diagonal) have higher values of connectivity. Moreover, all frequency bands except delta show higher values for off-diagonal entries corresponding to homologous ROIs (left frontal ROIs with right frontal ROIs, left parietal ROIs with right parietal etc.).

Finally, for all the bands, higher values of connectivity can be observed between left frontal ROIs and both left\right temporal ROIs, and reciprocally between right frontal ROIs and left\right temporal ROIs.

For matrices obtained with corrected AEC and PLI many of the higher connectivity values around the diagonal (neighboring ROIs) are absent. Although PLI seems to be noisier than AEC-c, visually they share a ‘four-block’ structure in the connectivity matrices for the theta to beta frequency bands. This ‘four-block’ structure corresponds to higher connectivity between parietal and occipital ROIs and between homologous parietal and occipital ROIs.

Matrices for the dPTE are not symmetric and by construction the entries of the lower part and the corresponding entries in the upper triangular part of the matrices add up to one. At least two different patterns can be visually discerned: (i) a preferential direction of information flow from posterior to anterior ROIs for higher frequency bands and (ii) the reversed pattern from anterior to posterior ROIs in the lower bands^8^.

Overall, the observation that patterns can be detected by averaging across subjects suggests that a common functional configuration is shared among subjects.

Figure S2: Connectivity matrices averaged across all epochs and all subjects. Each column represents a different frequency band (delta, theta, alpha, beta and gamma), and each row shows results for a different connectivity measure (AEC, AEC-c, i.e. AEC with leakage correction, PLI and dPTE). For the dPTE matrices each column i of a matrix represents the directed connectivity from ROI i to all other ROIs. Every matrix contains ranked values for visualization purposes. ROIs are obtained from the AAL atlas^9^, ordered from left to right hemisphere in the following way: indices from 1 to 39 represent left hemisphere (15 frontal, 6 parietal, 6 occipital and 12 temporal regions) while indices from 40 to 78 the right hemisphere (15 frontal, 6 parietal, 6 occipital and 12 temporal regions) (see also Table S2).

| ***Cortical regions of interest (ROIs)*** | | | |
| --- | --- | --- | --- |
| 1 | Gyrus Rectus (Left) | 40 | Gyrus Rectus (Right) |
| 2 | Olfactory Cortex (Left) | 41 | Olfactory Cortex (Right) |
| 3 | Superior frontal gyrus, orbital part (Left) | 42 | Superior frontal gyrus, orbital part (Right) |
| 4 | Superior frontal gyrus, medial orbital  (Left) | 43 | Superior frontal gyrus, medial orbital  (Right) |
| 5 | Middle frontal gyrus, orbital part (Left) | 44 | Middle frontal gyrus, orbital part (Right) |
| 6 | Inferior frontal gyrus, orbital part (Left) | 45 | Inferior frontal gyrus, orbital part (Right) |
| 7 | Superior frontal gyrus, dorsolateral  (Left) | 46 | Superior frontal gyrus, dorsolateral  (Right) |
| 8 | Middle frontal gyrus (Left) | 47 | Middle frontal gyrus (Right) |
| 9 | Inferior frontal gyrus, opercular part (Left) | 48 | Inferior frontal gyrus, opercular part (Right) |
| 10 | Inferior frontal gyrus, triangular part  (Left) | 49 | Inferior frontal gyrus, triangular part  (Right) |
| 11 | Superior frontal gyrus, medial (Left) | 50 | Superior frontal gyrus, medial (Right) |
| 12 | Supplementary motor area (Left) | 51 | Supplementary motor area (Right) |
| 13 | Paracentral lobule (Left) | 52 | Paracentral lobule (Right) |
| 14 | Precentral gyrus (Left) | 53 | Precentral gyrus (Right) |
| 15 | Rolandic operculum (Left) | 54 | Rolandic operculum (Right) |
| 16 | Postcentral gyrus (Left) | 55 | Postcentral gyrus (Right) |
| 17 | Superior parietal gyrus (Left) | 56 | Superior parietal gyrus (Right) |
| 18 | Inferior parietal, but supramarginal and angular gyri (Left) | 57 | Inferior parietal, but supramarginal and angular gyri (Right) |
| 19 | Supramarginal gyrus (Left) | 58 | Supramarginal gyrus (Right) |
| 20 | Angular gyrus (Left) | 59 | Angular gyrus (Right) |
| 21 | Precuneus (Left) | 60 | Precuneus (Right) |
| 22 | Superior occipital gyrus (Left) | 61 | Superior occipital gyrus (Right) |
| 23 | Middle occipital gyrus (Left) | 62 | Middle occipital gyrus (Right) |
| 24 | Inferior occipital gyrus (Left) | 63 | Inferior occipital gyrus (Right) |
| 25 | Calcarine fissure and surrounding cortex (Left) | 64 | Calcarine fissure and surrounding cortex (Right) |
| 26 | Cuneus (Left) | 65 | Cuneus (Right) |
| 27 | Lingual gyrus (Left) | 66 | Lingual gyrus (Right) |
| 28 | Fusiform gyrus (Left) | 67 | Fusiform gyrus (Right) |
| 29 | Heschl gyrus (Left) | 68 | Heschl gyrus (Right) |
| 30 | Superior temporal gyrus (Left) | 69 | Superior temporal gyrus (Right) |
| 31 | Middle temporal gyrus (Left) | 70 | Middle temporal gyrus (Right) |
| 32 | Inferior temporal gyrus (Left) | 71 | Inferior temporal gyrus (Right) |
| 33 | Temporal pole: superior temporal gyrus (Left) | 72 | Temporal pole: superior temporal gyrus (Right) |
| 34 | Temporal pole: middle temporal gyrus (Left) | 73 | Temporal pole: middle temporal gyrus (Right) |
| 35 | Parahippocampal gyrus (Left) | 74 | Parahippocampal gyrus (Right) |
| 36 | Anterior cingulate and paracingulate gyri (Left) | 75 | Anterior cingulate and paracingulate gyri (Right) |
| 37 | Median cingulate and paracingulate gyri (Left) | 76 | Median cingulate and paracingulate gyri (Right) |
| 38 | Posterior cingulate gyrus (Left) | 77 | Posterior cingulate gyrus (Right) |
| 39 | Insula (Left) | 78 | Insula (Right) |

Table S2: Cortical regions used in this study^9,10^.

# Identification rate using a parcellation scheme with 264 regions of interest

The identification rates using an atlas with 264 cortical regions^11^ are reported in Table S3 and Table S4. As a general result, identification rates increased when using the atlas with 264 ROIs compared with the one reported in the main manuscript (78 ROIs, see table 1). This is true when comparing the results for the pooled functional connectivity profiles, and also after the removal of the common pattern (using singular value decomposition (SVD)). Moreover, in general identification rates improved with the higher resolution atlas also when using individual frequency bands as a profile for the identification, both with the original data and after the removal of the common pattern (compare Tables 2 and S4).

|  | ***FC profile pooled across bands*** | |
| --- | --- | --- |
| FC | *original* | *SVD* |
| AEC | 48.4%*** | 71.9%*** |
| AEC-c | 32.8%*** | 43.7%*** |
| PLI | 7.8%*** | 28.1%*** |
| dPTE | 68.7%*** | 76.6%*** |

Table S3: Twin identification success rate using the global FCF or ECF based on different measures. For every subject the global FCF or ECF was obtained combining the subject’s FCF or ECF computed for the individual frequency band. The success rate for every FC and EC connectivity measure is reported: Amplitude Envelope Correlation without (AEC) and with correction (AEC-c), directed Phase Transfer Entropy (dPTE) and Phase Lag Index (PLI). Success rate based on the original data, as well as after removal of the common pattern across subjects (using SVD), are given. The asterisks represent the significant values after permutation testing: p-value ≤ 0.05(*), p-value ≤ 0.01(**) and p-value ≤ 0.001(***).

|  | **delta** | | **theta** | | **alpha** | | **beta** | | **gamma** | |
| --- | --- | --- | --- | --- | --- | --- | --- | --- | --- | --- |
| FC | *original* | *SVD* | *original* | *SVD* | *original* | *SVD* | *original* | *SVD* | *original* | *SVD* |
| AEC | 10.9%*** | 51.6%*** | 15.6%*** | 53.1%*** | 21.9%*** | 64.1%*** | 35.9%*** | 70.3%*** | 15.6%*** | 48.4%*** |
| AEC-c | 3.1% | 6.2%* | 10.9%*** | 14.1%*** | 15.6%*** | 28.1%*** | 21.9%*** | 34.4%*** | 7.8%** | 3.1% |
| PLI | 3.1% | 4.7% | 3.1% | 3.1% | 7.8%** | 15.6%*** | 10.9%*** | 14.1%*** | 9.4%*** | 6.2%* |
| dPTE | 6.2%* | 6.2%* | 14.1%*** | 15.6%*** | 37.5%*** | 48.4%*** | 39.1%*** | 48.4%*** | 26.6%*** | 31.2%*** |

Table S4: Twin identification success rates for different FC or EC measures in individual frequency bands: Amplitude Envelope Correlation without (AEC) and with correction (AEC-c), directed Phase Transfer Entropy (dPTE) and Phase Lag Index (PLI). The success rate based on the original data, as well as after removal of the common pattern across subjects (using SVD), are given. The asterisks represent the significant values after permutation testing: p-value ≤ 0.05(*), p-value ≤ 0.01(**) and p-value ≤ 0.001(***).

Bibliography

1. Stam, C. J., Nolte, G. & Daffertshofer, A. Phase lag index: Assessment of functional connectivity from multi channel EEG and MEG with diminished bias from common sources. *Hum Brain Mapp* **28,** 1178–1193 (2007).

2. Bruns, A., Eckhorn, R., Jokeit, H. & Ebner, A. Amplitude envelope correlation detects coupling among incoherent brain signals. *Neuroreport* **11,** 1509–1514 (2000).

3. Schreiber, T. Measuring information transfer. *Phys. Rev. Lett.* **85,** 461–464 (2000).

4. Lobier, M., Siebenhuhner, F., Palva, S. & Palva, J. M. Phase transfer entropy: A novel phase-based measure for directed connectivity in networks coupled by oscillatory interactions. *Neuroimage* **85,** 853–872 (2014).

5. Palus, M. & Stefanovska, A. Direction of coupling from phases of interacting oscillators: An information-theoretic approach. *Physical Review E* **67,** (2003).

6. Prokopenko, M. & Lizier, J. T. Transfer Entropy and Transient Limits of Computation. *Scientific Reports* **4,** (2014).

7. Otnes, R. K. & Enochson, L. *Digital time series analysis*. (John Wiley & Sons, 1972).

8. Hillebrand, A. *et al.* Direction of information flow in large-scale resting-state networks is frequency-dependent. *Proc. Natl. Acad. Sci. U.S.A.* **113,** 3867–3872 (2016).

9. Tzourio-Mazoyer, N. *et al.* Automated Anatomical Labeling of Activations in SPM Using a Macroscopic Anatomical Parcellation of the MNI MRI Single-Subject Brain. *Neuroimage* **15,** 273–289 (2002).

10. Gong, G. *et al.* Mapping anatomical connectivity patterns of human cerebral cortex using in vivo diffusion tensor imaging tractography. *Cereb. Cortex* **19,** 524–536 (2009).

11. Power, J. D. *et al.* Functional Network Organization of the Human Brain. *Neuron* **72,** 665–678 (2011).
